# Computer aided epitope design as a peptide vaccine component against Lassa virus

**DOI:** 10.6026/97320630013417

**Published:** 2017-12-31

**Authors:** Ar-Rafi Md. Faisal, Syed Hassan Imtiaz, Tasnim Zerin, Tania Rahman, Hossain Uddin Shekhar

**Affiliations:** 1 Department of Biochemistry and Molecular Biology, University of Dhaka, Dhaka-1000, Bangladesh; 2 Department of Genetic Engineering and Biotechnology, University of Dhaka, Dhaka-1000, Bangladesh;

**Keywords:** Lassa virus, envelope glycoprotein, epitope-based vaccine

## Abstract

Lassa virus (LASV) is an arena virus causing hemorrhagic fever and it is endemic in several regions of West Africa. The disease-causing virus records high mortality rate in endemic regions due to lack of appropriate treatment and prevention strategies. Therefore, it is of interest to design and develop viable vaccine components against the virus. We used the Lassa virus envelope glyco-proteins as a vaccine target to identify linear peptides as potential epitopes with immunogenic properties by computer aided epitope prediction tools. We report a T-cell epitope 'LLGTFTWTL' and a B-cell epitope 'AELKCFGNTAVAKCNE' with predicted potential immunogenicity for further in vivo and in vitro consideration.

## Background

Lassa virus is a single-stranded, enveloped, bi-segmented, ambisense RNA virus belonging to Arenaviridae virus family [1]. It causes severe forms of acute viral hemorrhagic fever known as Lassa hemorrhagic fever, endemic in regions of West Africa [2]. In West Africa, Lassa virus (LASV) causes as many as 300,000 infections and approximately 5,000 deaths per year [3]. This high rate of mortality in endemic areas, along with the absence of effective treatment and vaccination options, makes LASV an important pathogen to study. LASV was first identified in Lassa village, Borno State at the northeastern region of Nigeria in 1969 [4]. This zoonotic virus exhibits persistent, asymptomatic infection with profuse urinary virus excretion in Mastomys natalensis, the ubiquitous and highly commensal rodent host [2]. Human transmission occurs through food or household items contaminated with infected Mastomys rats' urine or feces. Personto- person transmission can occur through direct contact with the blood, urine, feces or other bodily secretions of infected person and indirect contact with environments contaminated with such fluids, which makes it highly susceptible for endemics or epidemics [5]. Sexual transmission has been reported and pregnant patients with Lassa fever results in spontaneous abortions [6]. Both sexes and all age groups of people appear to be affected by this virus and there is no epidemiological evidence supporting airborne spread between humans (WHO, 2017).

The incubation period of Lassa fever ranges from 6-21 days [7]. About 80% of people who become infected with LASV have no symptoms. 1 in 5 infections result in severe disease where the virus affects several organs such as liver, spleen and kidneys. The onset of the disease, when it is symptomatic, is usually gradual, starting with fever, general weakness, and malaise followed by sore throat, muscle pain, chest pain and in severe cases facial edema, fluid in the lung cavity, bleeding from the mouth, nose, vagina or gastrointestinal tract may develop [2]. Sensory-neural hearing loss (SNHL) is one of the common complications affecting as many as 25% of patients and rendering an estimated 1 to 2% of the population hearing impairment in areas with high rates of LASV infection [4]. Death usually occurs within 14 days of onset in fatal cases. Because of diverse and non-specific symptoms, it is often difficult to clinically diagnose Lassa fever and distinguish it from other viral hemorrhagic fevers such as Ebola virus disease, typhoid fever and yellow fever especially in the early course of the disease [8]. LASV outbreak first appeared in 1972 in Zorzor, Liberia [9]. According to WHO, there have been reports of re-emerged LASV infections followed by high mortality endemic outbreaks in Nigeria (2012); Nigeria, Benin, Togo, Sweden, Liberia (2016); Nigeria, Benin, Togo and Burkina Faso (2017) (WHO, 2017).

LASV genome contains two RNA segments coding for two proteins each. The larger segment is approximately 7.2kb and encodes a small zinc-binding protein regulating transcription, replication and RNA polymerase [10]. The smaller segment is approximately 3.4kb encoding the nucleoprotein and the envelope glycoprotein [11]. Even though the mortality caused by LASV was first reported in almost 45 years ago, little effort has been made to cure and/or prevent its detrimental effects till date. Treatment with the antiviral drug ribavirin seems to be effective for Lassa fever but it must be administered in the first week of illness for optimal efficacy [12]. However, even early commencement of ribavirin therapy seems not to offer protection against development of SNHL [4]. The best immediate prospect to control this disease in endemic areas lies with the use of a vaccine.

Innovative stride towards the development of LASV vaccine was made earlier in this century and it was reported that trials on primates were successful with the vaccine, but there were no reports on the human trials or further advancement of that approach [13]. Currently, there is no effective vaccine against Lassa fever (WHO, 2017). Developing inactivated vaccines would be an option but they are less effective and need regular booster injections [14]. Therefore, developing peptide vaccines would be more effective to restrain the spread or new emerging epidemics of Lassa virus [15]. Hence, we performed analysis to predict T and B cell epitope based peptides with LASV envelope glycoprotein having known immunity [16]. Various immuno-informatics approaches were used to find effective T and B cell epitopes for the design and development of a viable vaccine against LASV.

## Methodology

**Figure 1:**
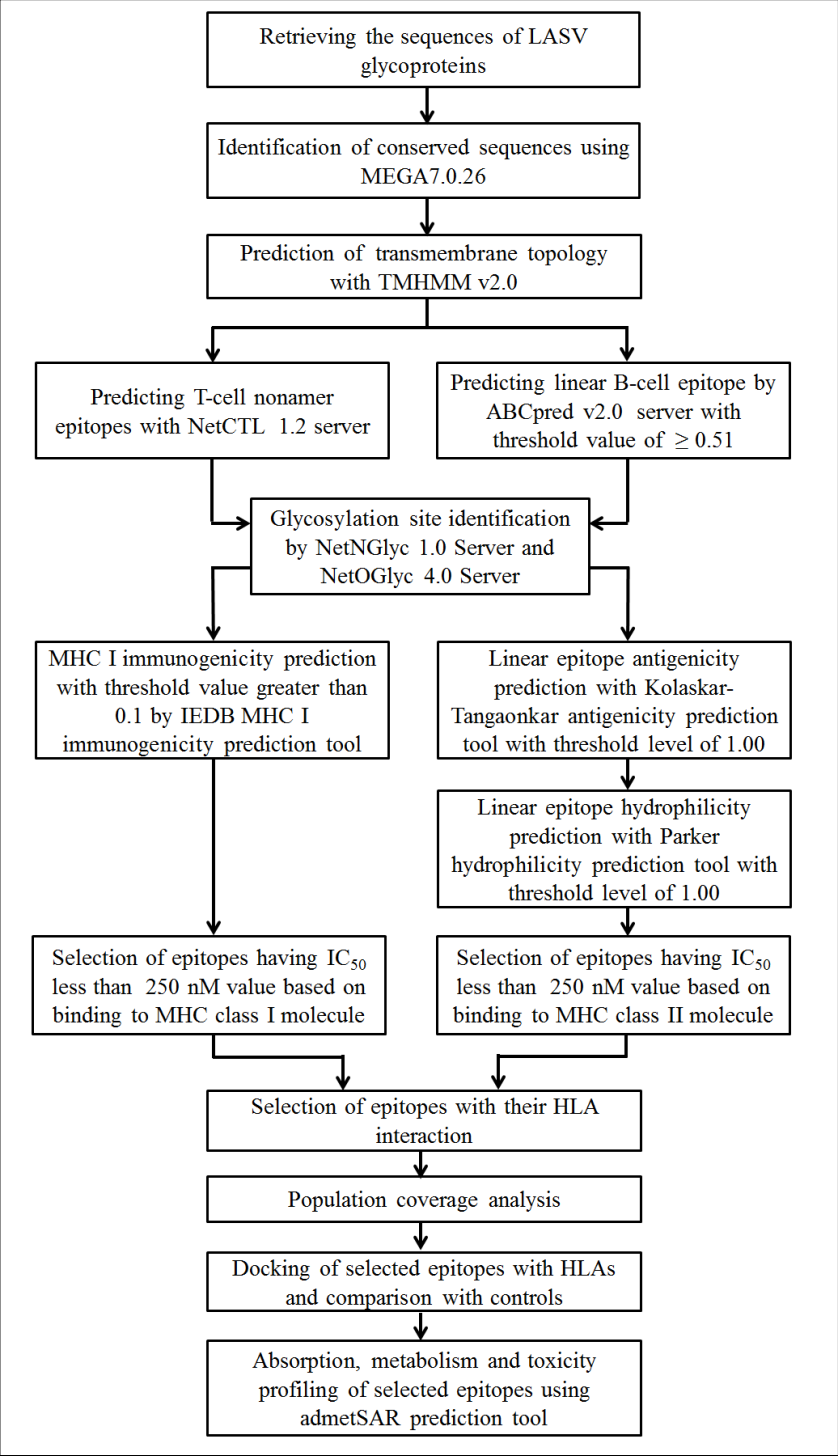
A workflow for the design of epitope as a peptide vaccine component against LASV.

An outline of the methodology used in this study shown in Figure 1.

### LASV protein sequences

The 12 sequences for glyco-proteins of different isolates of LASV were retrieved from NCBI (National center for biotechnology information) database. These sequences were obtained from different strains of LASV at different time frame (like at 1987, 1989, 2002, 2010, 2011 and 2017) in different endemic regions of Africa.

### Multiple Sequence Alignment (MSA)

Retrieved sequences were subjected to multiple sequence alignment using MEGA 7.0.26 software package (http://www.megasoftware.net). The CLUSTALW algorithm along with 1000 bootstrap value and other default parameters were adopted to fabricate the alignment. The sequences were analyzed to recognize the immunologically relevant regions that were achieved by predicting epitopic peptides. An amino acid stretch must be of a minimum length for being considered as an epitope that we are aiming to design. Due to representative length of peptide that binds to HLA molecules, nonamers were selected as the minimum length of the conserved sequences for the prediction of epitope-based peptide in the current study.

### Transmembrane Topology Analysis

The conserved regions from the glyco-proteins were analyzed to distinguish their soluble and membrane regions. Each selected conserved sequence was subjected to transmembrane topology prophecy using TMHMM v0.2 server to identify the inner, outer and transmembrane portion with high degree of accuracy [17]. It should be noted that vaccine components can be effectively designed with epitopes at the exposed regions of the membrane.

### T-Cell Epitope prediction in conserved regions

The cytotoxic T lymphocyte (CTL) epitopes from the conserved peptides were predicted using the NetCTL 1.2 server, which is based on neural network architecture and predicts candidate epitopes based on the processing of the peptides in vivo [18]. During analysis, threshold, sensitivity and specificity were set at 0.75, 0.8 and 0.97 respectively. A combined algorithm integrating MHC class I binding, transporter of antigenic peptides (TAP) transport efficiency and proteasomal cleavage prediction was involved to predict a total/overall score. Weight on C-terminal cleavage 0.15 and weight on TAP (transporter of antigenic peptides) transport efficiency was determined 0.05. The best epitope candidates were chosen based on a combined score. Primarily selected epitopes were undertaken for further analysis. For note, these epitopes were nonamer in length.

### Linear B-Cell Epitope Prediction from the Conserved Sequences

Linear B-cell epitope prediction was done with ABCpred v2.0 server, which uses artificial neural network [19]. This webserver is based on recurrent neural network (machine based technique) using fixed length patterns. The epitope length was selected as sixteen-mer. Epitopes, which had crossed the threshold level of 0.51, were selected for further analysis. The epitopes were also checked with Bepipred linear epitope prediction tool by IEDB [20].

### Prediction of glycosylation sites

NetNGlyc 1.0 Server and NetOGlyc 4.0 Server were used to check the glycoprotein sequences respectively. The epitopes, which had no glycosylation site, were considered for further analysis.

### MHC Class I and T-Cell Epitope Interaction

The immune epitope database and analysis resource (IEDB) MHC class I binding prediction tool was used to calculate IC50 (half maximal inhibitory concentration) values for peptides binding to specific MHC I molecules. This tool is based on stabilized matrix method-based prediction (SMM) [21]. Epitopes taken from NetCTL analysis and having no glycosylation site were used here. T-cell epitopes were then analyzed with IEDB MHC I immunogenicity scores. The parameters for immunogenicity detection (TAP score, proteasomal score and IC50 values) were normalized on a scale of 0 to 1 and were given a weighted score to prioritize the parameters in order to determine immunogenicity [22].

### MHC Class II and B-Cell Epitope Interaction

Prior to predicting epitopes interacting with MHC class II, they were first checked for their antigenic properties like antigenicity and hydrophilicity by Kolaskar-Tangaonkar antigenicity prediction tool and Parker hydrophilicity prediction tool respectively [23, 24]. The epitopes that didn't possess any glycosylation sites were analyzed in these tools and those, which crossed the threshold level of 1.00 for each of the properties, were further checked to have association with MHC II alleles. IEDB MHC II binding tool was used to check the association with MHC II alleles [25]. Likewise in MHC I binding interaction analysis, SMM was also used to analyze HLA relationship with epitopes. The epitopes, which had highest interactions, were selected for further analysis.

### Population Coverage

Selected T-cell and B-cell epitopes were used for population coverage analysis using population coverage tool from the IEDB analysis resource [26]. The allele frequency of the interacting HLA alleles was used to measure the population coverage for the corresponding epitope.

### Molecular Docking Study of HLA-Epitope Interaction

#### Retrieving 3D Structure of HLAs

The 3D structure of HLA-A*02:01 (PDB ID: 3UTQ) and HLADRB1* 01:01 (PDB ID: 1AQD) were downloaded from the Protein Data Bank (PDB) database. Prior to docking, all the water molecules were removed from the 3D structure of epitope free HLA-A*02:01 and HLA-DRB1*01:01.

#### Designing of the 3D Epitope Structure

The 3D structures of epitopes were designed using the PEPFOLD peptide structure prediction server at the Ressource Parisienne en Bioinformatique Structurale Mobyle Portal [27].

#### HLA-Epitope Binding Prediction

The AutoDock tool from the Molecular Graphics Laboratory software package (version 1.5.6) was employed for docking purpose. Both the proteins and ligands (epitopes) files were converted to PDBQT format for using in this docking study. The grid/ space box center for B-cell epitope and HLA-DRB1*01:01 was set at 32.4206, -0.6947 and 6.2001 Å in the x, y and z axes respectively, so that the epitope could bind at the binding groove of HLA-DRB1*01:01. The grid/ space box center for T-cell epitope and HLA-A*02:01 was set at 11.9650, 0.3823 and 16.3061 Å in the x, y and z axes respectively, so that the epitope could bind at the binding groove of HLA-A*02:01. The size was set at 104.8110, 83.3764 and 133.0247 Å in the x-, y- and z-dimensions, respectively.

All the analysis was done at 1.00 Å spacing. The number of outputs was set at 10, while the exhaustiveness was kept at the default 8.00. AutoDock Vina program was used to perform the actual docking based on these parameters [28].

Outputs were again visualized in the PYMOL molecular graphics system and the best output was selected based on higher binding energy. The binding affinity or the docking results were collected in .csv format which opens in Microsoft excel. The non-bond interaction between proteins and the ligands were visualized using Accelrys Discovery Studio Visualizer (version 2017 R2). The types of bonding, their distance and the categories of bonding were also visualized. The hydrophobic and ionizable receptor surfaces were determined using this software.

#### Absorption, Metabolism and Toxicity Profiling of Candidate Peptide Vaccines

Absorption, metabolism and toxicity profiles of the peptide epitopes were predicted using "admetSAR" prediction server (http://lmmd.ecust.edu.cn/admetsar1/).

## Results

### Identification of conserved regions in antigen sequences

A total of 12 sequences of glycoproteins from different isolates of LASV have been retrieved from GenBank at NCBI (Table S1). The CLUSTALW program in MEGA software generated several conserved sequences with varying lengths. A total of 10 conserved sequences were found.

### Transmembrane Topology Determination

TMHMM v2.0 prediction analysis revealed that 6 (out of 10) conserved regions of envelope glycoproteins fulfilled the criteria of outer membrane characteristics (Table S2).

### Immunogenicity Prediction of T-Cell Epitope

NetCTL tool predicted 66 possible interactions with MHC supertypes, which were, determined with a combinatorial score of TAP score, MHC I binding score, proteasomal cleavage score and antigen processing score.

### Linear B-Cell Epitope Prediction

**Figure 2:**
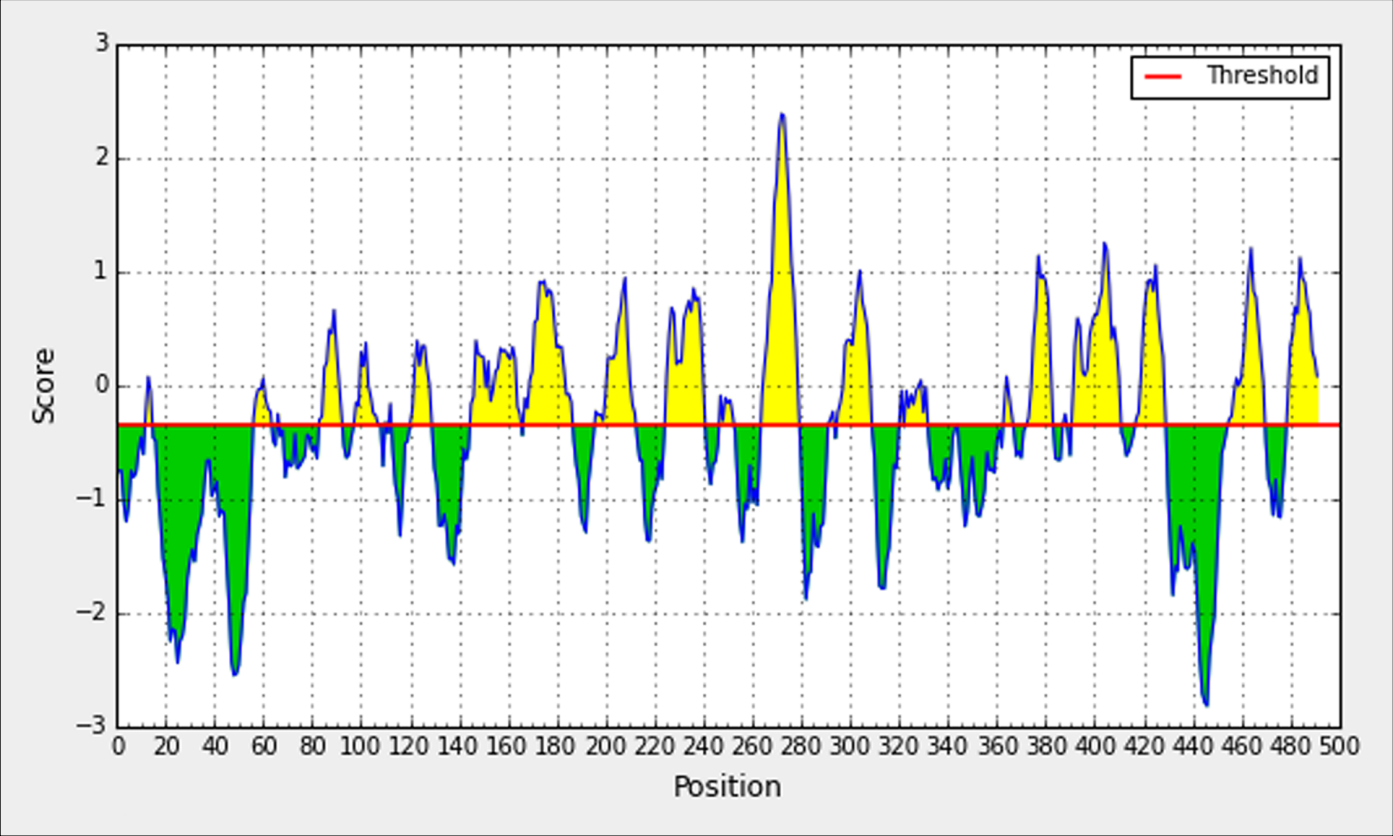
Bepipred linear epitope prediction tool depicting a B cell epitope.

ABCpred server was used to predict sixteen-mer epitopes from the selected conserved sequences. This tool found 29 epitopes. The Bepipred linear epitope prediction tool was used to analyze conserved regions for epitope identification. This is followed by accuracy crosscheck for epitope prediction using ABCpred (Figure 2).

### Glycosylation site prediction

**Figure 3:**
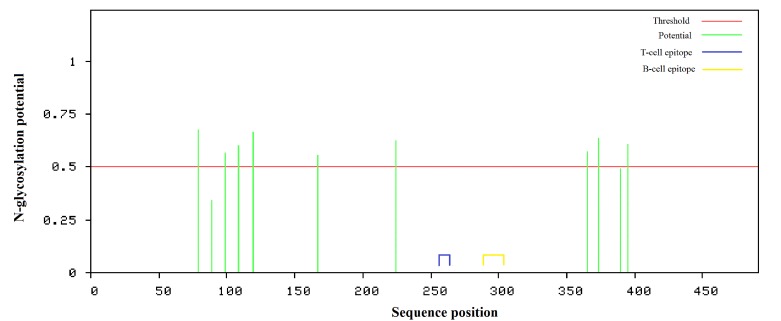
Schematic diagram of the N-glycosylation sites of LASV glycoprotein (491 amino acids). Amino acids residue numbers 79, 89, 99, 109, 119, 167, 224, 365, 373, 390 and 395 are predicted to be N-glycosylation sites. The blue and the yellow area indicate the predicted T-cell candidate epitope 'LLGTFTWTL' region (258-266) and predicted B-cell candidate epitope 'AELKCFGNTAVAKCNE' region (288-303). Both the epitopes do not possess any glycosylation site.

11 N-glycosylation sites were found but there were no sites for Oglycosylation in the target protein sequence (Figure 3). Among the 66 nonamer T cell epitopes and 29 sixteen-mer B-cell epitopes, 51 T cell epitopes and 22 B-cell epitopes did not possess any glycosylation sites (Table S3, Table S5).

### MHC Class I and T-Cell Epitope Interaction

All the 51 T-cell epitopes were then analyzed with an stabilization matrix method in IEDB MHC-I binding prediction tool having IC50 score of less than 250 nM (IC50< 250 nM) matching 81 possible MHC-I allele interactions with 17 different T-cell epitopes (Table S4). Top 6 epitopes were selected having IEDB MHC I antigenicity score over 0.2 (Table 1). They were further checked with population coverage and molecular docking.

### MHC Class II and B-Cell Epitope Interaction

The 22 B cell epitopes that were selected by ABCpred server and did not contain any glycosylation site within them were checked with Kolaskar-Tongaonkar antigenicity prediction tool and Parker hydrophilicity prediction tool for predicting the antigenicity and the hydrophilicity of the epitopes (Table S5). 7 epitopes crossed the threshold level of 1.00 for each of the properties (antigenicity and hydrophilicity) and were selected for further analysis. IEDB MHC II binding prediction tool then provided interactions between these epitopes and MHC II alleles. Interactions, which had IC50 value less than 250 nM, were then selected (Table S6). Finally three 16-mer epitopes were selected based on prominent interactions with MHC II alleles (Table 2). These epitopes were further checked with population coverage and molecular docking.

### Population Coverage

**Figure 4:**
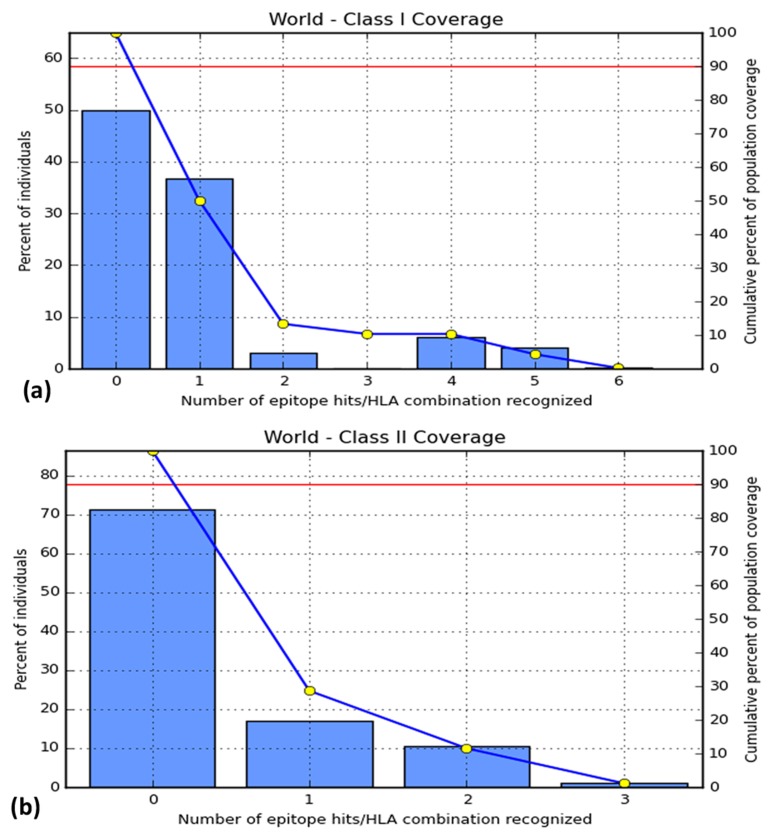
Worldwide population coverage of (a) T-cell epitopes and (b) B-cell epitopes with MHC class I alleles and MHC class II alleles respectively. Bar '0' indicates the percentage of people not elucidating immune response by the epitopes. In the figures (a) and (b), bar '1' indicates the highest interaction showed by the epitopes 'LLGTFTWTL' and 'AELKCFGNTAVAKCNE' respectively.

For the epitopes of class I MHC molecules, Europe showed a coverage of 59%, North America showed almost 50%, West Africa showed 22.14% and the worldwide coverage was 50.04% (Table S7). 'LLGTFTWTL' showed highest coverage among the T-cell epitopes, which is 38% (Figure 4a). For the epitope of class II MHC molecules, epitopes provided the coverage of 36.78% in Europe, which was the highest, and worldwide coverage was just at 28.63%. West Africa showed population coverage of 12.69% (Table S8). 'AELKCFGNTAVAKCNE' showed 18% coverage, which is the highest in case of B-cell epitopes (Figure 4b).

### Molecular Docking Study of HLA-Epitope Interaction

**Figure 5:**
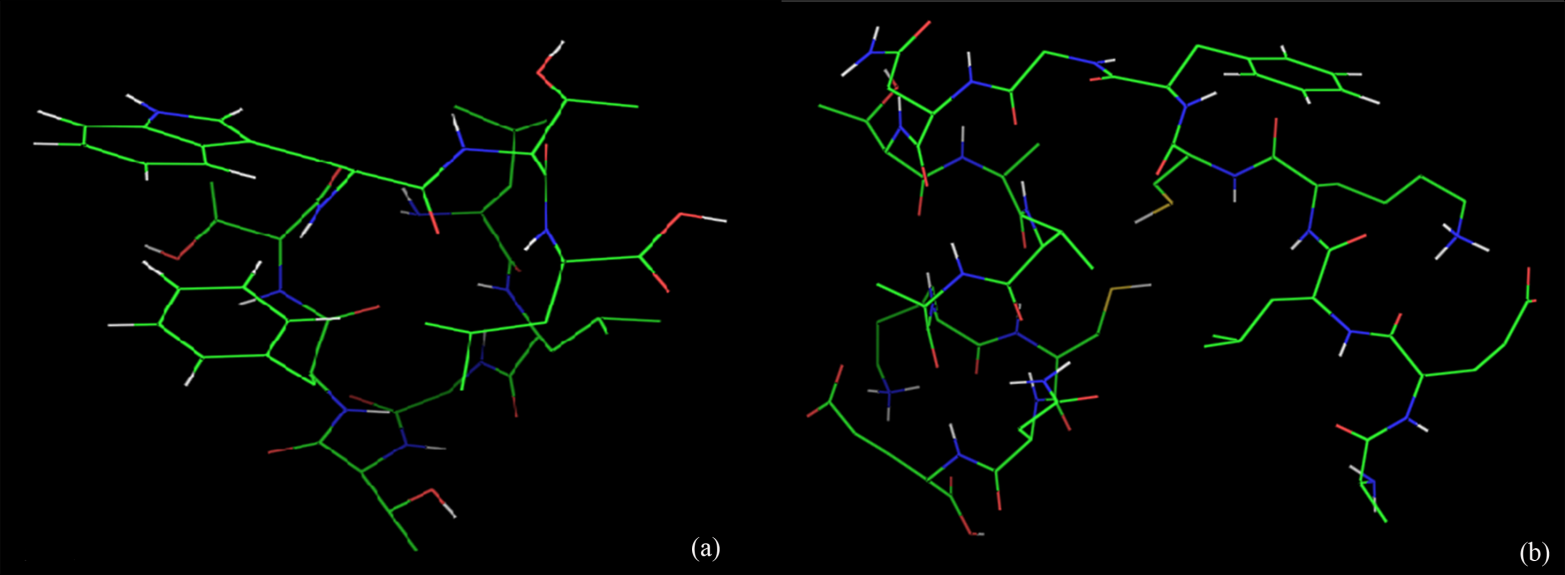
The epitopes from the conserved regions of LASV glycoproteins. (a) T-cell epitope 'LLGTFTWTL' (b) B-cell epitope 'AELKCFGNTAVAKCNE'

Binding models of the good epitopes ('LLGTFTWTL' and 'AELKCFGNTAVAKCNE' epitopes; Figure 5) to its specific HLA molecules were completed using AutoDock Vina.

**Figure 6:**
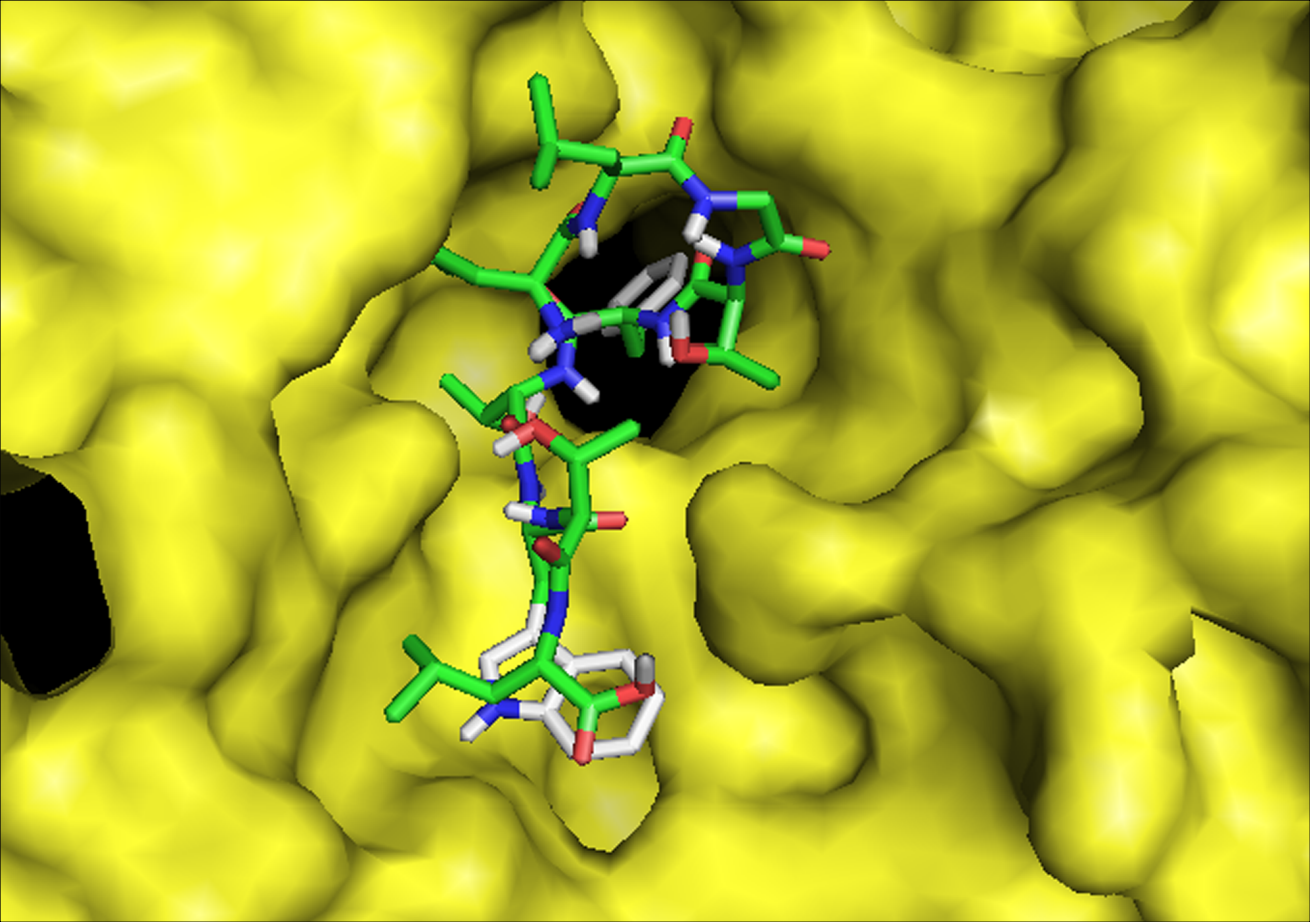
Molecular docking peptide 'LLGTFTWTL' to the binding grooves of MHC class I molecule, HLA-A*02:01. Binding energy was -10.5 kcal/mol.

T cell epitope 'LLGTFTWTL' bound to the binding groove of HLA-A*02:01 with a binding energy of -10.5 kcal/mol is shown (Figure 6). The binding energy of control peptide 'FLNKDLEVDGHFVTM' to the binding grooves of the HLAA* 02:01 were found to be -11.4 kcal/mol (PDB ID: 4U6Y). This comparison corrects that T-cell epitope 'LLGTFTWTL' binds with HLA-A*02:01 maintaining almost the same binding energy. The peptide ligand 'LLGTFTWTL' interacts with leu 9, arg 6, thr 8, trp 7 and thr 6 of the HLA-A*02:01 protein molecule. There are electrostatic, hydrophobic and conventional hydrogen bond formed between the ligand and the HLA-A*02:01 protein (Table S1).

**Figure 7:**
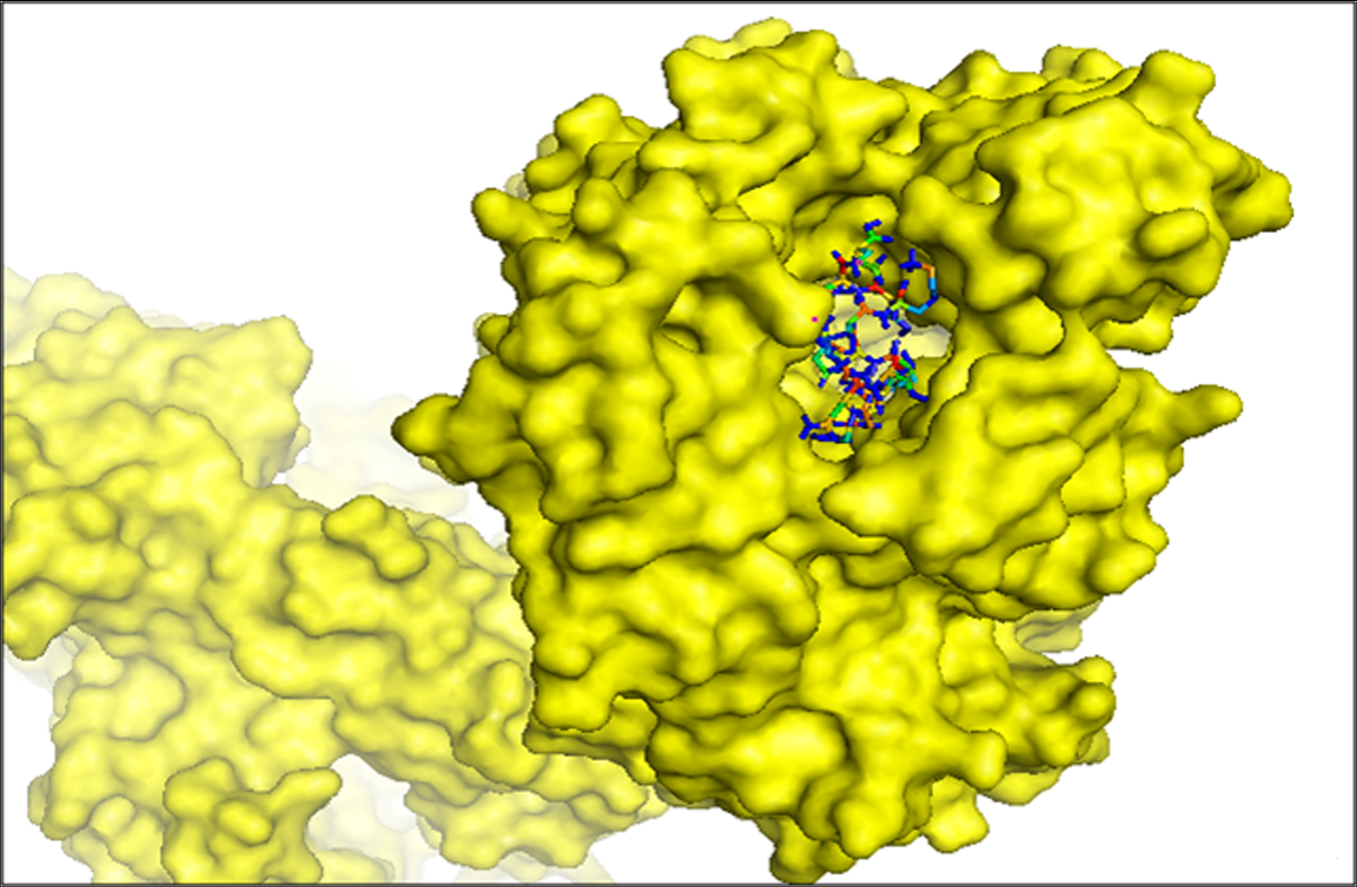
Molecular docking of candidate epitopic peptides to MHC class II molecule, HLA-DRB1*01:01. The bindings of predicted peptide, 'AELKCFGNTAVAKCNE' to the binding grooves of HLA-DRB1*01:01 and binding energy was found to be -14.1 kcal/mol.

The binding energy for B cell epitope 'AELKCFGNTAVAKCNE' to the binding groove of HLA-DRB1*01:01, was estimated to be - 14.1 kcal/mol (Figure 7). The peptide ligand 'AELKCFGNTAVAKCNE' forms electrostatic bond, conventional hydrogen bond, carbon hydrogen bond, alkyl and pi-alkyl bond with HLA-DRB1*01:01 (Figure S2). The key amino acids of the HLA-DRB1*01:01 protein that are situated at the active site and interacts with the ligand are Glu 2, Glu 16, Asn 8, Asn 15, Lys 13, Lys 4, Cys 5, Ala 10, Val 11 and phe 6. Altogether, it can be concluded that both the epitopes showed satisfactory binding affinities with respective HLA allele.

### Absorption, Metabolism and Toxicity Profiling of Candidate Peptide Vaccines

Through "admetSAR" we performed the screening of absorption, metabolism and toxicity profiles of these two epitope-based vaccine candidates and found that both the projected epitopes were predicted to be impermeable to blood brain barrier, noninhibitor of P-glycoprotein and non-inhibitor of renal organic cation transporter (Table 3). Moreover, they were predicted to be non-mutagenic (according to the result of AMES toxicity prediction), non-carcinogenic and non-inhibitor of a variety of CYP450 enzymes. Both the epitopes had acute oral toxicity level III (Category III includes compounds with LD50 values greater than 500mg/kg but less than 5000mg/kg) indicating large amount will be required to cause toxicity, which in turn predicted the non-toxic characteristics of the epitopes. Both the predicted epitopes showed good characteristics with prediction analyses and are predicted to be the potential candidates for in vitro and in vivo analysis for further consideration.

## Discussion

LASV is an endemic virus. It holds a catastrophic potential to destroy the medical scenario in several regions of the world. So, developing a vaccine as a prevention method against LASV is important. Therefore, it is of interest to investigate potential vaccine candidates from LASV glycoprotein. Glycoprotein remains in the outer membrane portion of the virus and it triggers the initial action for viral infection or entry into the host. Vaccine development using glycoprotein targets is highly viable in the context of virus infection [29]. A pipeline for developing envelope glycoprotein epitope based vaccine is found to be highly efficient against LASV infection.

A previous study (Verma et al. 2015) was completed using glycoprotein (Accession Number NP_694870.1) without considering other isolates specific to LASV endemic regions in different time frame [30]. The available 12 LASV glycoprotein sequences from different isolates at different time frame in endemic regions were retrieved for this study and conserved regions among them were identified and illustrated. Viruses tend to mutate faster and therefore, it is important that the candidate epitopes should be in the highly conserved region so that the vaccine designed remain active and provide protection for longer period and against a longer range of virus strains. This, we believe, would generate more acceptable epitope(s) that should be effective universally. Moreover, the epitopes are also needed to be outside the membrane as immune cells activity is modulated by the outer membrane portion as they encounter with the virus during the initial period of infection. Thus, the conserved sequences that fulfill the outer membrane characteristics were used for further analyses that were neglected in the earlier study [30].

Our interest in designing B cell epitopes is because of its role to induce the production of antibodies synthesized by B cells and mediates its effector functions [31]. However, over time, humoral response from memory B cells can easily be overcome by surge of antigens whereas cell mediated immunity (T cell immunity) often elicits lasting immunity [32]. Cytotoxic T lymphocytes (CTL) restrict the spread of pathogens (like virus) by recognizing and killing infected cells and secreting specific antiviral cytokines, which in turn elicit strong immune response [33]. Hence, T-cell epitopes were predicted for the design.

NetCTL tool was used to predict cytotoxic T cell epitopes as it integrates prediction of peptide MHC class I binding, proteasomal C terminal cleavage, TAP transport efficiency. The best 66-epitope candidates were chosen based on a combined score. While projecting the B-cell epitopes, ABCpred server was used and it was found that 29 epitopes cross the threshold level of 0.51. Moreover, BepiPred was also used to predict B cell epitopes with other tools so that everybody can compare the results showed by different prediction tools. This study using different tools will make the study more concrete and increase the probability of epitope as a favorable candidate for further wet-lab verifications.

Another important concern with glycoprotein is to elicit immunity against protein antigen attached with sugar moiety. Protein glycosylation have crucial influence on uptake and proteolytic processing of protein antigens by sterically blocking the action of proteases [34]. This in turn can affect MHC presentation and subsequent immune response [35]. For example, gp120 subunit of HIV envelope glycoprotein is heavily Nglycosylated which facilitates viral escape from the host immune system by constraining proteolytic processing of the protein antigen required for antigen presentation and cytotoxic T-cell priming [36]. Moreover, the N-glycans can also block access of neutralizing antibodies to critical epitopes [35]. Thus, protein sequences were checked for glycosylation site. Only the nonglycosylated epitopes (51 out of 66 T cell epitopes and 22 out of 29 B-cell epitopes) were taken for further analyses. This approach was not considered in previous reports.

Interaction analysis between different MHC supertypes/HLA alleles and epitopes in IEDB, SMM method was used among others (like NetMHCpan and CombLib) in this study. This is because both input and output are quantitative in these tools. The output is easy to interpret and specific computational strategies are built to handle experimental noise [21].

Predicted T cell epitope ('LLGTFTWTL') showed adequate MHC I antigenicity score (0.41022). It is also found to interact with MHC-I alleles with IC50 score of less than 250 nM having high population coverage of 38%. Moreover, this T cell epitope bound to the binding groove of HLA-A*02:01 with a binding energy of - 10.5 kcal/mol similar to the control peptide's binding affinity (- 11.4 kcal/mol).

However, the B cell epitope (288-303), 'AELKCFGNTAVAKCNE' showed adequate score like ABCpred Score (0.95), antigenicity score (1.04) and hydrophilicity score (2.431). It also showed dominant interactions with MHC II alleles with IC50 score of less than 250 nM having high population coverage of 18%. The binding energy for the epitope at the binding groove of HLADRB1* 01:01, was estimated to be -14.1 kcal/mol. Meulen et al. (2004) reported a highly conserved region having an overlap of two CD4+T-cell epitopes defined by amino acids (289-301) and (282-294) with this epitope (288-303). The epitope between residues 289-301 is 100% conserved in known arena viruses implying its evolutionary and functional importance [37]. Thus, the significance of the epitope for further in vivo and in vitro consideration is established.

## Conclusion

Identification of epitopes in LASV is non-trivial towards the development of a peptide based vaccine formulation. We report a T-cell epitope 'LLGTFTWTL' and a B-cell epitope 'AELKCFGNTAVAKCNE' with predicted immunogenicity for further in vivo and in vitro consideration.

## Conflict of Interest

The authors declare no conflict of interests.

## Supplementary material

Data 1

## Figures and Tables

**Table 1:** MHC class I specific nonamer epitopes with antigenicity score (over 0.2) having IC50 values less than 250 nM

Sequence	Antigenicity score	HLA class I alleles
LLGTFTWTL	0.41022	HLA-A*02:01, HLA-C*12:03, HLA-C*03:03, HLA-B*15:02
RLLGTFTWT	0.34068	HLA-C*03:03, HLA-C*12:03, HLA-C*14:02, HLA-A*02:06
KHDEEFCDM	0.28311	HLA-C*12:03, HLA-C*05:01, HLA-C*14:02, HLA-C*07:02
RWMLIEAEL	0.24721	HLA-C*12:03, HLA-C*03:03, HLA-C*14:02
SQRTRDIYI	0.2373	HLA-C*12:03, HLA-A*30:01
MLRLFDFNK	0.23554	LA-C*12:03, HLA-A*30:01, HLA-C*12:03, HLA-A*03:01, HLA-A*31:01, HLA-A*11:01, HLA*68:01

**Table 2:** MHC class II specific 16 mer B-cell linear epitopes with alleles, ABCpred score, antigenicity score and hydrophilicity score

Sequence	MHC II HLA	ABCpred Score	Antigenicity (IEDB)	Hydrophilicity (IEDB)
AELKCFGNTAVAKCNE	HLA-DRB1*07:01, HLA DRB1*01:01, HLA-DRB1*04:04, HLA-DQA1*05:01/DQB1*03:01	0.95	1.04	2.431
AEAQMSIQLINKAVNA	HLA-DRB4*01:01, HLA-DRB1*01:01, HLA-DRB1*04:04, HLA-DRB1*11:01	0.82	1.025	1.331
YKGVYELQTLELNMET	HLA-DRB1*01:01, HLA-DRB1*04:05, HLA-DPA1*02:01/DPB1*01:01, HLA-DPA1*03:01/DPB1*04:02	0.55	1.015	1.181

**Table 3:** ADMET predicted profile classification for predicted T cell epitope'‘LLGTFTWTL' and B cell epitope 'AELKCFGNTAVAKCNE'

Absorption	Predicted T cell epitope 'LLGTFTWTL'		Predicted B cell epitope 'AELKCFGNTAVAKCNE'	
Model	Result	Probability	Result	Probability
Blood-Brain Barrier	BBB-	0.8154	BBB-	0.9273
P-glycoprotein Inhibitor	Non-inhibitor	0.8743	Non-inhibitor	0.949
Renal Organic Cation Transporter	Non-inhibitor	0.9127	Non-inhibitor	0.9444
Metabolism	Predicted T cell epitope 'LLGTFTWTL'		Predicted B cell epitope 'AELKCFGNTAVAKCNE'	
Model	Result	Probability	Result	Probability
CYP450 1A2 Inhibitor	Non-inhibitor	0.8878	Non-inhibitor	0.8708
CYP450 2C9 Inhibitor	Non-inhibitor	0.8367	Non-inhibitor	0.8816
CYP450 2D6 Inhibitor	Non-inhibitor	0.9006	Non-inhibitor	0.8891
CYP450 2C19 Inhibitor	Non-inhibitor	0.8146	Non-inhibitor	0.8031
CYP450 3A4 Inhibitor	Non-inhibitor	0.9172	Non-inhibitor	0.8443
Toxicity	Predicted T cell epitope 'LLGTFTWTL'		Predicted B cell epitope 'AELKCFGNTAVAKCNE'	
Model	Result	Probability	Result	Probability
AMES Toxicity	Non AMES toxic	0.8561	Non AMES toxic	0.7732
Carcinogens	Non-carcinogens	0.8709	Non-carcinogens	0.8836
Acute Oral Toxicity	III	0.5729	III	0.649
