# Supplementary material for: Computer aided epitope design as a peptide vaccine component against Lassa virus
Source: Bioinformation. 2017 Dec 31;13(12):417–29. doi: 10.6026/97320630013417 (PMC5767920; doi:10.6026/97320630013417)
Supplement: Data 1 [file 97320630013417S1.pdf]

BIOINFORMATION
Discovery at the interface of physical and biological sciences

Open access

www.bioinformation.net

Volume 13(12)

Hypothesis

## Supplementary Material:

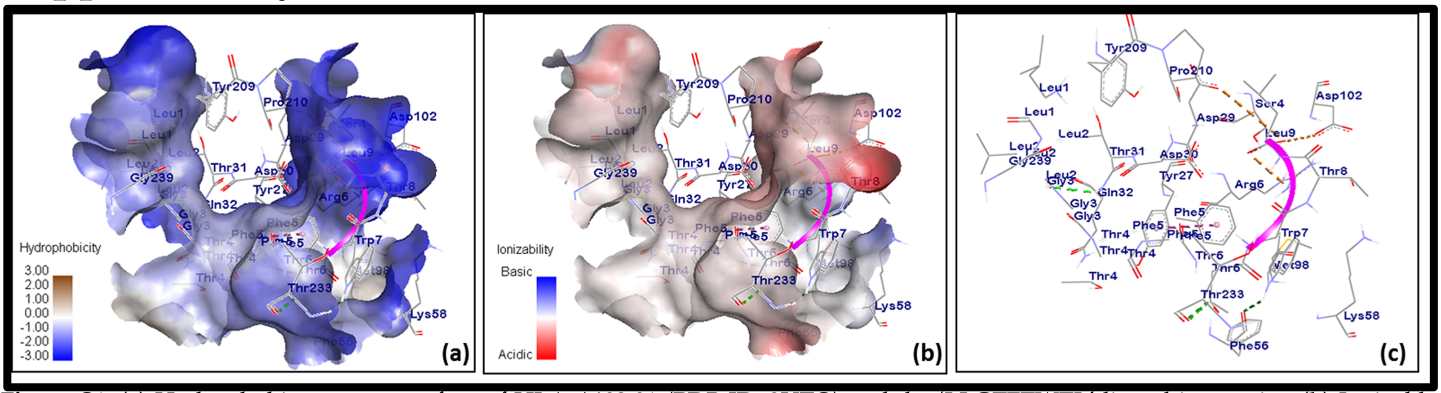


**Figure S1: (a)** Hydrophobic receptor surface of HLA-A*02:01 (PDB ID: 3UTQ) and the 'LLGTFTWTL' ligand interaction **(b)** Ionizable receptor surface of HLA-A*02:01 (PDB ID: 3UTQ) and the interaction of 'LLGTFTWTL' ligand and **(c)** Non-bond interaction between HLA-A*02:01 (PDB ID: 3UTQ) and 'LLGTFTWTL' ligand.

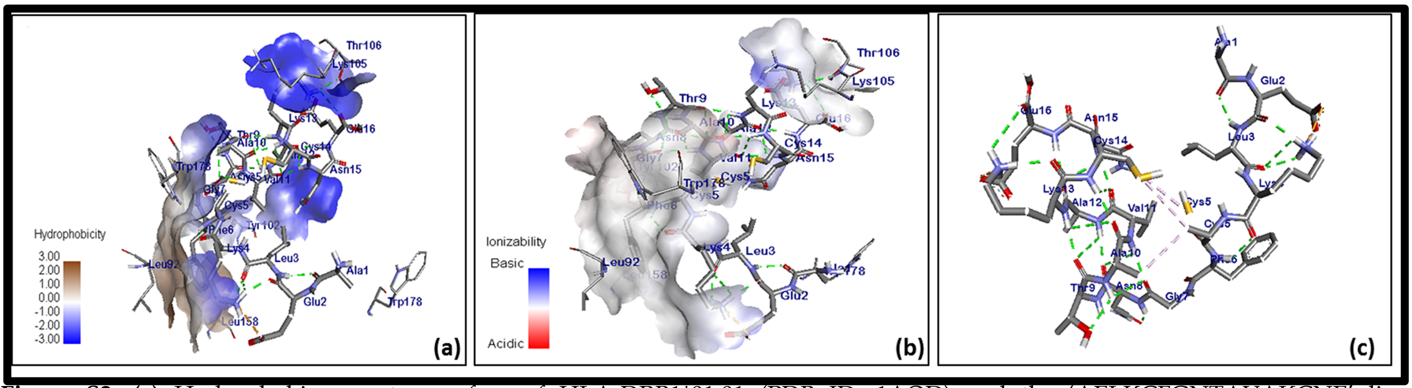


**Figure S2: (a)** Hydrophobic receptor surface of HLA-DRB1*01:01 (PDB ID: 1AQD) and the 'AELKCFGNTAVAKCNE' ligand interaction **(b)** Ionizable receptor surface of HLA-DRB1*01:01 (PDB ID: 1AQD) and the interaction of 'AELKCFGNTAVAKCNE' ligand **(c)** Non-bonded interaction between HLA-DRB1*01:01 (PDB ID: 1AQD) and the 'AELKCFGNTAVAKCNE' ligand.

**Table S1:** Glycoprotein sequences from different strains of LASV at different time frame in different endemic regions

| LASV glycoprotein sequences | Accession number |
|---|---|
| LASVGP_1987 | AAA46283.1 |
| LASVGP_1989 | AAA46286.1 |
| LASVGP_Josiah_Sep2002 | NP_694870.1 |
| LASVGP_Josiah_Dec2010 | ADY11068.1 |
| LASVGP_recombinant_Josiah_Dec2010 | ADY11070.1 |
| LASVGP_Josiah_Aug2011_Sierra Leone | AEY85213.1 |
| LASVGP_Josiah_Jan2017_Sierra Leone | APT69664.1 |
| LASVGP_Josiah_Jan2017_Sierra Leone | APT69609.1 |
| LASVGP_Jan2017_Guinea | APT69607.1 |
| LASVGP_Josiah_Jan2017_Sierra Leone | APT69605.1 |
| LASVGP_Josiah_Jan2017_Sierra Leone | APT69592.1 |
| LASVGP_Jan2017_Guinea | APT69572.1 |

**Table S2:** Transmembrane topology analysis of LASV glycoprotein conserved regions using TMHMM v2.0 server

| Conserved regions | Nature |
|---|---|
| 1-27 | Inner membrane + Transmembrane |
| 29-37 | Transmembrane |
| 59-131 | Outer membrane |
| 133-173 | Outer membrane |
| 175-220 | Outer membrane |
| 222-375 | Outer membrane |
| 380-409 | Outer membrane |
| 411-427 | Outer membrane |
| 428-461 | Inner + Transmembrane |
| 463-478 | Inner membrane |



©2017

**Table S3:** Results from non-glycosylated T-cell epitope prediction with MHC class I binding score, processing score, proteasomal cleavage score, TAP score interaction along with super-type information is shown.

| Sequence | MHC super-type | MHC score | processing score | proteasomal cleavage score | TAP score | Total score |
|---|---|---|---|---|---|---|
| ITEMLQKEY | A1 | 0.5415 | 2.2993 | 0.9549 | 2.818 | 2.5834 |
| | B62 | 0.2637 | 0.5234 | 0.9549 | 2.818 | 0.8076 |
| LSIPNFNQY | A1 | 0.3242 | 1.3764 | 0.9728 | 3.015 | 1.6731 |
| | A26 | 0.3454 | 0.927 | 0.9728 | 3.015 | 1.2237 |
| | B58 | 0.4724 | 1.0647 | 0.9728 | 3.015 | 1.3614 |
| | B62 | 0.4597 | 0.9126 | 0.9728 | 3.015 | 1.2093 |
| LSQRTRDIY | A1 | 0.2816 | 1.1957 | 0.3009 | 2.985 | 1.3901 |
| | B62 | 0.3411 | 0.6772 | 0.3009 | 2.985 | 0.8716 |
| NWDCIMTSY | A1 | 0.2562 | 1.0876 | 0.8691 | 3.002 | 1.368 |
| FSRPSPIGY | A1 | 0.2354 | 0.9996 | 0.7407 | 2.87 | 1.2542 |
| | B58 | 0.3128 | 0.705 | 0.7407 | 2.87 | 0.9596 |
| | B62 | 0.5028 | 0.9981 | 0.7407 | 2.87 | 1.2527 |
| LRDIMGIPY | A1 | 0.214 | 0.9087 | 0.9175 | 2.936 | 1.1931 |
| | B27 | 0.2951 | 0.7845 | 0.9175 | 2.936 | 1.0689 |
| MRMAWGGSY | A1 | 0.1321 | 0.5611 | 0.8509 | 3.138 | 0.8456 |
| | B27 | 0.5452 | 1.4493 | 0.8509 | 3.138 | 1.7339 |
| | B62 | 0.4623 | 0.9178 | 0.8509 | 3.138 | 1.2023 |
| SLYKGVYEL | A2 | 0.7619 | 1.1357 | 0.9778 | 1.224 | 1.3435 |
| | B39 | 0.3597 | 1.1517 | 0.9778 | 1.224 | 1.3596 |
| | B62 | 0.2898 | 0.5752 | 0.9778 | 1.224 | 0.7831 |
| LLGTFTWTL | A2 | 0.7622 | 1.1362 | 0.9783 | 0.947 | 1.3303 |
| RMAWGGSYI | A2 | 0.6266 | 0.934 | 0.9571 | 0.953 | 1.1252 |
| | B62 | 0.4864 | 0.9656 | 0.9571 | 0.953 | 1.1568 |
| RLFDFNKQA | A2 | 0.5849 | 0.8719 | 0.8557 | -0.112 | 0.9946 |
| CIMTSYQYL | A2 | 0.5237 | 0.7806 | 0.1666 | 0.968 | 0.854 |
| IISTFHLSI | A2 | 0.4681 | 0.6978 | 0.6683 | 0.736 | 0.348 |
| ALINDQLIM | A2 | 0.4559 | 0.6796 | 0.7948 | 0.678 | 0.8327 |
| | A26 | 0.2836 | 0.7611 | 0.7948 | 0.678 | 0.9143 |
| | B62 | 0.3129 | 0.6213 | 0.7948 | 0.678 | 0.7744 |
| MAWGGSYIA | A2 | 0.4616 | 0.688 | 0.8474 | -0.132 | 0.8086 |
| RLLGTFTWT | A2 | 0.4924 | 0.734 | 0.5216 | -0.292 | 0.7977 |
| MTMPLSCTK | A3 | 0.6468 | 1.2174 | 0.9195 | 0.696 | 1.3901 |
| MLRLFDFNK | A3 | 0.5932 | 1.1164 | 0.8549 | 0.441 | 1.2667 |
| LINDQLIMK | A3 | 0.5415 | 1.0191 | 0.7773 | 0.657 | 1.1686 |
| QMSIQLINK | A3 | 0.5685 | 1.0699 | 0.3017 | 0.749 | 1.1526 |
| RTRDIYISR | A3 | 0.4475 | 0.8422 | 0.6413 | 1.707 | 1.0238 |
| MLQKEYMER | A3 | 0.3859 | 0.7262 | 0.9385 | 1.352 | 0.9346 |
| LMSIISTFH | A3 | 0.424 | 0.798 | 0.0903 | -0.26 | 0.7986 |
| QYEAMSCDF | A24 | 0.577 | 1.2286 | 0.6609 | 2.809 | 1.4682 |
| VYELQTLEL | A24 | 0.5585 | 1.1893 | 0.9583 | 1.107 | 1.3877 |
| | B39 | 0.2581 | 0.8262 | 0.9583 | 1.107 | 1.0246 |
| RWMLIEAEL | A24 | 0.9396 | 1.1489 | 0.9371 | 1.52 | 1.3655 |
| TFHLSIPNF | A24 | 0.5158 | 1.0982 | 0.4659 | 2.765 | 1.3064 |
| GYCLTRWML | A24 | 0.4916 | 1.0467 | 0.5185 | 1.019 | 1.1755 |
| EFCDMLRLF | A24 | 0.4277 | 0.9107 | 0.1669 | 2.34 | 1.0527 |
| | A26 | 0.4232 | 1.1357 | 0.1669 | 2.34 | 1.2777 |
| AWGGSYIAL | A24 | 0.3554 | 0.7567 | 0.8072 | 1.218 | 0.9387 |
| EGKDTPGGY | A26 | 0.5752 | 1.5435 | 0.9526 | 2.5060 | 1.8117 |
| DCIMTSYQY | A26 | 0.5009 | 1.3442 | 0.2989 | 0.185 | 1.3983 |
| TTWEDHCQF | A26 | 0.1784 | 0.4788 | 0.9761 | 2.805 | 0.7654 |
| SPIGYLGLL | B58 | 0.5342 | 1.2039 | 0.9761 | 2.805 | 1.4906 |
| ISRRLLGTF | B8 | 0.3758 | 1.2834 | 0.8407 | -0.075 | 1.4058 |
| LINKAVNAL | B7 | 0.3062 | 0.5907 | 0.782 | 0.944 | 0.7552 |
| | B8 | 0.2316 | 0.7908 | 0.374 | 2.595 | 0.9766 |
| YISRRLLGT | B8 | 0.2604 | 0.8894 | 0.0430 | -0.592 | 0.8662 |
| RRLLGTFTW | B58 | 0.3064 | 0.6906 | 0.9146 | 1.0960 | 0.8826 |
| SIQLINKAV | B8 | 0.1972 | 0.6733 | 0.4906 | 0.5 | 0.7719 |
| | B27 | 0.5307 | 1.4109 | 0.9784 | 1.409 | 1.628 |
| QRLKAEAQM | B27 | 0.5114 | 1.3594 | 0.8823 | 0.551 | 1.5192 |
| KHDEEFCDM | B44 | 0.2765 | 0.6853 | 0.1647 | 0.944 | 0.7572 |


©2017

BIOINFORMATION
Discovery at the interface of physical and biological sciences

Open access

| | | | | | | |
|---|---|---|---|---|---|---|
| TLELNMETL | B44 | 0.6825 | 1.6912 | 0.3874 | 0.745 | 1.7866 |
| HDEEFCDML | B39 | 0.256 | 0.8197 | 0.6187 | 0.513 | 0.9381 |
| | B39 | 0.21 | 0.6725 | 0.9134 | 0.76 | 0.8475 |
| AEAQMSIQL | B39 | 0.2054 | 0.6577 | 0.3779 | 1.081 | 0.7684 |
| EEFCDMLRL | B44 | 0.747 | 1.8512 | 0.3779 | 1.081 | 1.9619 |
| RDIYISRRL | B44 | 0.3093 | 0.7664 | 0.4343 | 0.97 | 0.88 |
| TEMLQKEYM | B44 | 0.2782 | 0.6895 | 0.609 | 0.222 | 0.792 |
| VANGVLQTF | B62 | 0.3842 | 0.7628 | 0.9593 | 0.904 | 0.9519 |
| MSIISTFHL | B58 | 0.6342 | 1.4293 | 0.9397 | 1.159 | 1.6282 |
| | B58 | 0.5985 | 1.3489 | 0.9048 | 2.565 | 1.6128 |
| LQTFMRMAW | B62 | 0.4276 | 0.8488 | 0.2256 | 2.72 | 1.0186 |
| SQRTRDIYI | B62 | 0.3315 | 0.6581 | 0.5659 | 0.76 | 0.781 |

**Table S4:** HLA class I specific T-cell epitopes with antigenicity score having $IC_{50}$ < 250 nM.

| Sequence | Antigenicity score | HLA class I alleles |
|---|---|---|
| LLGTFTWTL | 0.41022 | HLA-A*02:01, HLA-C*12:03, HLA-C*03:03, HLA-B*15:02 |
| RLLGTFTWT | 0.34068 | HLA-C*03:03, HLA-C*12:03, HLA-C*14:02, HLA-A*02:06 |
| KHDEEFCDM | 0.28311 | HLA-C*12:03, HLA-C*05:01, HLA-C*14:02, HLA-C*07:02 |
| RWMLIEAEL | 0.24721 | HLA-C*12:03, HLA-C*03:03, HLA-C*14:02 |
| SQRTRDIYI | 0.2373 | HLA-C*12:03, HLA-A*30:01 |
| GYCLTRWML | 0.1422 | HLA-C*14:02, HLA-C*07:02, HLA-C*12:03, HLA-B*15:02, HLA-C*03:03, HLA-A*23:01 |
| HDEEFCDML | 0.11262 | HLA-C*12:03, HLA-C*07:02, HLA-B*15:02, HLA-C*05:01 |
| TTWEDHCQF | 0.11152 | HLA-C*14:02, HLA-A*32:01, HLA-C*03:03, HLA-C*12:03 |
| LRDIMGIPY | 0.10786 | HLA-C*12:03, HLA-C*14:02, HLA-C*08:02 |
| MLRLFDFNK | 0.23554 | HLA-C*12:03, HLA-A*30:01, HLA-C*12:03, HLA-A*03:01, HLA-A*31:01, HLA-A*11:01, HLA-A*68:01 |
| MRMAWGGSY | 0.16191 | HLA-C*12:03, HLA-C*06:02, HLA-C*07:01, HLA-C*14:02, HLA-B*15:02, HLA-B*27:05, HLA-A*30:02, HLA-A*29:02 |
| RMAWGGSYI | 0.15871 | HLA-C*03:03, HLA-A*02:01, HLA-C*12:03, HLA-C*14:02, HLA-B*15:02, HLA-A*02:06, HLA-A*32:01 |
| LMSIISTFH | 0.15525 | HLA-C*03:03, HLA-C*12:03, HLA-C*14:02 |
| RRLLGTFTW | 0.17626 | HLA-C*12:03, HLA-A*32:01, HLA-B*58:01, HLA-C*03:03, HLA-B*27:05, HLA-C*07:02 |
| MSIISTFHL | 0.17026 | HLA-B*15:02, HLA-B*58:01, HLA-A*68:02, HLA-C*12:03, HLA-C*03:03, HLA-A*02:06 |
| LSQRTRDIY | 0.19748 | HLA-C*12:03, HLA-C*03:03, HLA-B*15:02, HLA-C*14:02, HLA-A*30:02 |
| RTRDIYISR | 0.1809 | HLA-A*31:01, HLA-A*30:01, HLA-C*12:03, HLA-C*14:02, HLA-A*68:01 |

**Table S5:** Results from non-glycosylated B-cell epitope prediction using the ABCpred server v2.0. Antigenicity and hydrophilicity were calculated using the IEDB antigen sequence properties tool.

| Rank | Sequence | Starting number | ABCpred Score | Antigenicity (IEDB) | Hydrophilicity (IEDB) |
|---|---|---|---|---|---|
| 01 | AELKCFGNTAVAKCNE | 288 | 0.95 | 1.04 | 2.431 |
| 02 | DQLIMKNHLRDIMGIP | 347 | 0.90 | 0.999 | 0.125 |
| 03 | LSDSEGKDTPGGYCLT | 266 | 0.90 | 1.007 | 3.375 |
| 04 | TEMLQKEYMERQGKTP | 412 | 0.89 | 0.946 | 3.106 |
| 05 | EEFCDMLRLFDFNKQA | 307 | 0.88 | 1.006 | 0.738 |
| 06 | WGGSYIALDSGRGNWD | 196 | 0.87 | 0.957 | 1.875 |
| 07 | HLSIPNFNQYEAMSCD | 141 | 0.87 | 1.027 | 1.625 |
| 08 | LGTFTWTLSDSEGKDT | 259 | 0.83 | 0.963 | 2.569 |
| 09 | TFMRMAWGGSYIALDS | 190 | 0.83 | 0.984 | 0.081 |
| 10 | MSIISTFHLSIPNFNQ | 134 | 0.83 | 1.025 | -0.431 |
| 11 | AEAQMSIQLINKAVNA | 328 | 0.82 | 1.025 | 1.331 |
| 12 | QRTRDIYISRRLLGTF | 247 | 0.81 | 1.008 | 0.619 |
| 13 | TAVAKCNEKHDEEFCD | 296 | 0.80 | 1.023 | 3.95 |
| 14 | KDTPGGYCLTRWMLIE | 272 | 0.78 | 1.006 | 0.656 |
| 15 | YISRRLLGTFTWTLSD | 253 | 0.76 | 1.018 | -0.25 |
| 16 | CLTRWMLIEAELKCFG | 279 | 0.75 | 1.055 | -1.106 |
| 17 | DHCQFSRPSPIGYLGL | 229 | 0.75 | 1.067 | 0.925 |
| 18 | NQYEAMSCDFNGGKIS | 148 | 0.73 | 0.981 | 3.006 |
| 19 | PLSCTKNNSHHYIMVG | 83 | 0.65 | 1.047 | 1.519 |
| 20 | DFNKQAIQRLKAEAQM | 317 | 0.63 | 0.986 | 2.131 |
| 21 | RPSPIGYLGLLSQRTR | 235 | 0.62 | 1.032 | 0.931 |
| 22 | YKGVYELQTLELNMET | 62 | 0.55 | 1.015 | 1.181 |

**Table S6:** MHC class II specific B-cell epitopes having predicted $IC_{50}$ less than 250 nM is listed.

| Sequence | MHC II HLA |
|---|---|
| AELKCFGNTAVAKCNE | HLA-DRB1*07:01, HLA-DRB1*01:01, HLA-DRB1*04:04, HLA-DQA1*05:01/DQB1*03:01 |



BIOMEDICAL INFORMATICS

©2017

| | |
|---|---|
| LSDSEGKDTPGGYCLT | Nil |
| HLSIPNFNQYEAMSCD | HLA-DRB1*01:01 |
| AEAQMSIQLINKAVNA | HLA-DRB4*01:01, HLA-DRB1*01:01, HLA-DRB1*04:04, HLA-DRB1*11:01 |
| PLSCTKNNSHHYIMVG | Nil |
| YKGVYELQTLELNMET | HLA-DRB1*01:01, HLA-DRB1*04:05, HLA-DPA1*02:01/DPB1*01:01, HLA-DPA1*03:01/DPB1*04:02 |
| TAVAKCNEKHDEEFCD | Nil |

**Table S7:** Population coverage of class I MHC specific T-cell epitopes

| Population/area | Class I | | |
|---|---|---|---|
| | Coverage | Average hit | pc90 |
| Central Africa | 18.78% | 0.36 | 0.12 |
| East Africa | 23.39% | 0.31 | 0.13 |
| East Asia | 38.61% | 0.45 | 0.16 |
| Europe | 59.02% | 1.15 | 0.24 |
| North Africa | 34.05% | 0.69 | 0.15 |
| North America | 49.7% | 0.72 | 0.2 |
| Northeast Asia | 26.32% | 0.43 | 0.14 |
| Oceania | 27.89% | 0.44 | 0.14 |
| South America | 28.08% | 0.44 | 0.14 |
| South Asia | 21.92% | 0.54 | 0.13 |
| Southeast Asia | 23.07% | 0.29 | 0.13 |
| Southwest Asia | 33.69% | 0.61 | 0.15 |
| West Africa | 22.14% | 0.33 | 0.13 |
| West Indies | 32.19% | 0.32 | 0.15 |
| World | 50.04% | 0.88 | 0.2 |

**Table S8:** Population coverage of class II MHC specific B-cell epitopes

| Population/area | Class II | | |
|---|---|---|---|
| | Coverage | Average hit | pc90 |
| Central Africa | 15.2% | 0.17 | 0.12 |
| Central America | 12.49% | 0.14 | 0.11 |
| East Africa | 12.76% | 0.17 | 0.11 |
| East Asia | 17.28% | 0.28 | 0.12 |
| Europe | 36.78% | 0.54 | 0.16 |
| North Africa | 27.63% | 0.3 | 0.14 |
| North America | 31.49% | 0.46 | 0.15 |
| Northeast Asia | 10.91% | 0.14 | 0.11 |
| Oceania | 2.67% | 0.03 | 0.1 |
| South America | 13.17% | 0.17 | 0.12 |
| South Asia | 36.27% | 0.47 | 0.16 |
| Southeast Asia | 7.13% | 0.08 | 0.11 |
| Southwest Asia | 14.43% | 0.17 | 0.12 |
| West Africa | 12.69% | 0.17 | 0.11 |
| West Indies | 26.71% | 0.37 | 0.14 |
| World | 28.63% | 0.41 | 0.14 |

ISSN 0973-2063 (online) 0973-8894 (print)

Bioinformation 13(12): 426-429 (2017)
BIOMEDICAL INFORMATICS
©2017
